# Effects of Alcohol on the Diseases of Europeans in India

**Published:** 1857-03

**Authors:** 


### EDITORIAL.

*Effects of Alcohol on the Diseases of Europeans in India.*

In the *Indian Annals of Medical Science*, for April, 1856, Mr. Waring has given some very interesting facts in relation to the prevalence of diseases among the different classes of Europeans in India. From them we quote the following in relation to the use and influence of alcoholic drinks:—

In the Madras Presidency, in 1849, there were six regiments of European troops, numbering 5,710 men. They were classified by Mr. Waring as follows:—Teetotallers 450, temperate 4,318, intemperate 942. The following table shows the actual amount and kind of disease suffered by these several classes during the year 1849:—

| DISEASES. | TETOTALLERS, 450. | | | TEMPERATE, 4318. | | | INTEMPERATE, 942. | | |
|---|---|---|---|---|---|---|---|---|---|
| | Attacks. | Deaths. | Ratio. | Attacks. | Deaths. | Ratio. | Attacks. | Deaths. | Ratio. |
| Fevers | 141 | 1 | 31,33 | 768 | 1 | 17,78 | 190 | 2 | 20,16 |
| Cholera | | | | 17 | 13 | 0,39 | 7 | 6 | 0,74 |
| Dysentery | 52 | 3 | 11,55 | 344 | 31 | 7,96 | 112 | 15 | 11,88 |
| Diarrhœa | 5C | 1 | 11,11 | 348 | 4 | 8,05 | 108 | | 11,46 |
| Other Diseases Stomach & Bowels | 23 | | 5,11 | 337 | 6 | 7,80 | 112 | 2 | 11,88 |
| Hepatitis | 26 | | 5,77 | 249 | 16 | 5,76 | 96 | 2 | 10,19 |
| Diseases of Lungs | 43 | | 9,55 | 478 | 17 | 11,06 | 113 | 4 | 11,99 |
| Do. of Brain | 14 | | 3,11 | 108 | 1 | 2,50 | 82 | 5 | 8,70 |
| Rheumatism | 27 | | 6,00 | 487 | | 11,27 | 143 | 1 | 15,18 |
| Venereal Diseases | 94 | | 20,88 | 1414 | 1 | 35,06 | 447 | | 50,63 |
| Other Diseases | 119 | | 26,44 | 1464 | 10 | 33,89 | 584 | 5 | 61,98 |
| Total, | 589 | 5 | 130,88 | 6114 | 100 | 141,59 | 2024 | 42 | 214,86 |

These results are rendered more obvious by the following table viz:—

| CLASS. | Whole Number. | No. of Attacks. | Deaths. | Ratio of Attacks. | Ratio of Deaths. | Ratio of Deaths to Attacks. |
|---|---|---|---|---|---|---|
| Teetotallers, | 450 | 589 | 5 | 130,88 | 1,11 | 0,84 |
| Temperate, | 4318 | 6114 | 100 | 141,59 | 2,31 | 1,63 |
| Intemperate, | 940 | 2024 | 42 | 214,86 | 4,45 | 2,07 |

Nothing could demonstrate more clearly the influence of alcoholic beverages on the prevalence of sickness and mortality than these figures. We hope our readers will note particularly the figures relating to *Cholera* and diseases of the *Lungs*.

